# Tumors that mimic asbestos-related mesothelioma: time to consider a genetics-based tumor registry?

**DOI:** 10.3389/fgene.2014.00151

**Published:** 2014-05-30

**Authors:** Brent D. Kerger, Robert C. James, David A. Galbraith

**Affiliations:** ^1^Cardno ChemRisk, LLCAliso Viejo, CA, USA; ^2^ToxStrategiesTallahassee, FL, USA; ^3^Cardno ChemRisk, LLCSan Francisco, CA, USA

**Keywords:** germ cell tumors, synovial sarcoma, pericardial mesothelioma, mullerian tissue cancers, chromosomal instability, human, asbestos

## Abstract

The diagnosis of mesothelioma is not always straightforward, despite known immunohistochemical markers and other diagnostic techniques. One reason for the difficulty is that extrapleural tumors resembling mesothelioma may have several possible etiologies, especially in cases with no meaningful history of amphibole asbestos exposure. When the diagnosis of mesothelioma is based on histologic features alone, primary mesotheliomas may resemble various primary or metastatic cancers that have directly invaded the serosal membranes. Some of these metastatic malignancies, particularly carcinomas and sarcomas of the pleura, pericardium and peritoneum, may undergo desmoplastic reaction in the pleura, thereby mimicking mesothelioma, rather than the primary tumor. Encasement of the lung by direct spread or metastasis, termed pseudomesotheliomatous spread, occurs with several other primary cancer types, including certain late-stage tumors from genetic cancer syndromes exhibiting chromosomal instability. Although immunohistochemical staining patterns differentiate most carcinomas, lymphomas, and mestastatic sarcomas from mesotheliomas, specific genetic markers in tumor or somatic tissues have been recently identified that may also distinguish these tumor types from asbestos-related mesothelioma. A registry for genetic screening of mesothelioma cases would help lead to improvements in diagnostic criteria, prognostic accuracy and treatment efficacy, as well as improved estimates of primary mesothelioma incidence and of background rates of cancers unrelated to asbestos that might be otherwise mistaken for mesothelioma. This information would also help better define the dose-response relationships for mesothelioma and asbestos exposure, as well as other risk factors for mesothelioma and other mesenchymal or advanced metastatic tumors that may be indistinguishable by histology and staining characteristics.

## Introduction

By far, the most extensively investigated cause of mesothelioma is asbestos exposure (Sporn and Roggli, 2004). Asbestos occurs in two mineralogic forms: commercially used amphiboles (e.g., commercially available amosite and crocidolite) and chrysotile. Amosite and crocidolite are considerably more persistent in tissue than is chrysotile, which disappears from lung tissue rapidly (Bernstein et al., 2005, 2010, 2011). This difference in biopersistence is believed to be the one of reasons that mesothelioma incidence rates are much higher in those who used or manufactured products with amosite and crocidolite, when compared to those exposed to only chrysotile (Churg, 1998; Hodgson and Darnton, 2000; Yarborough, 2006).

Although there is some debate regarding the potency gradient between chrysotile and asbestos fiber types, most agree that amphiboles are more toxic on a fiber-to-fiber basis (Hodgson and Darnton, 2000, 2010; Berman and Crump, 2008a,b). Some epidemiologic observations suggest that the incidence of mesothelioma caused by asbestos exposure peaked between about 1990 and 2010, because the highly potent amphibole exposures were largely curtailed in the 1960s and the typical latency period of 20–40 years since first exposure has now transpired (Hemminki and Li, 2003; Hillard et al., 2003; Price and Ware, 2004; Weill et al., 2004; Burdorf et al., 2007; Teta et al., 2008; Harding and Darnton, 2010). Epidemiologic observations also suggest a much lower, if not zero, mesothelioma potency for the more prominent chrysotile exposures from various sources and products in subsequent years (Hodgson and Darnton, 2000, 2010; Berman and Crump, 2003, 2008a,b; Goodman et al., 2004; Laden et al., 2004; Gibbs and Berry, 2008; Sichletidis et al., 2008). If this is true, it suggests that sometime in the near future there may be a substantial decline in the number of asbestos-induced mesothelioma cases. Consequently, there will be a greater need to clinically distinguish and better understand the etiology of mesothelioma cases unrelated to asbestos exposure.

While only perhaps 10–20% of pleural mesothelioma cases in the past decade are reportedly due to causes other than asbestos exposure (Sporn and Roggli, 2004), the risk factors for peritoneal mesothelioma are far more diverse, with less than half of the cases in recent case series explained by heavy amphibole exposures (Neumann et al., 2001; Hillard et al., 2003; Weill et al., 2004; Reid et al., 2005; Bofetta and Stayner, 2006; Bofetta, 2007; Larson et al., 2007; Magnani et al., 2007; Gibbs and Berry, 2008). Pericardial mesothelioma is far less common than the pleural and peritoneal forms and has no strong or consistent association with asbestos exposure (Papi et al., 2005; Luk et al., 2008). In addition, unlike pleural mesothelioma, peritoneal and pericardial mesotheliomas often occur in younger individuals with no known or substantial source of past asbestos exposure (Sporn and Roggli, 2004; Papi et al., 2005; Luk et al., 2008). Recent reviews acknowledge that asbestos is not the sole cause of pleural and peritoneal mesothelioma, with erionite and ionizing radiation being established causes and the relatively low frequency of mesothelioma among more highly exposed individuals indicating that genetic susceptibility factors and/or co-carcinogens may play an important role (Neri et al., 2005; Testa et al., 2011; Jasani and Gibbs, 2012; Jean et al., 2012; Tallet et al., 2013).

Rates of peritoneal mesothelioma have stayed relatively flat in developed countries during the past two decades, in contrast to the dramatic rise in pleural mesothelioma rates from about the 1960s through at least the mid-1990s that was associated with asbestos use in prior decades (Hemminki and Li, 2003; Leigh, 2003; Weill et al., 2004; Burdorf et al., 2007; Teta et al., 2008; Moolgavkar et al., 2009; Harding and Darnton, 2010). This divergence suggests that asbestos is not likely to be among the current major causes of peritoneal mesothelioma. In fact, there is emerging evidence that some of the tumors initially diagnosed as peritoneal mesotheliomas are in actuality a cancer with another origin. Peritoneal tumors may easily be misclassified as they often result from metastatic disease originating in other organs and are typically discovered at late stages when such tumors may exhibit mixed pathological characteristics that do not closely resemble the original tumor cell type (Pass et al., 2005; Burdorf et al., 2007). It has even been suggested that peritoneal mesothelioma in younger individuals (e.g., under age 50) may represent a distinctly different disease entity; one that is more curable and more likely related to gonadal cancers, and so, not plausibly attributed to asbestos exposure (Weill et al., 2004; Hodgson et al., 2005; Larson et al., 2007).

**Figure 1:**
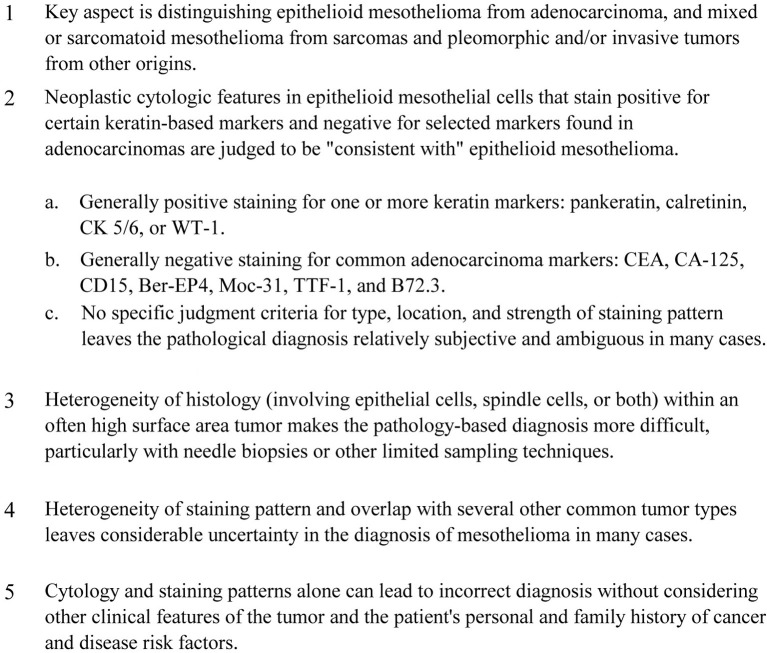
**Difficulties with diagnosis of primary mesothelioma using only cytology and immunohistochemical staining pattern**.

Because mesothelioma lacks a unique and distinctive pattern of histological features, the diagnosis is often tentative and based primarily on a defined pattern of staining with immunohistochemical markers (Ordonez, 1998; Cappello and Barnes, 2001). While most tumor presentations can be distinguished by the presence/absence of these specific immunohistochemical markers, in many cases a definitive staining pattern is lacking. The difficulties sometimes encountered with mesothelioma diagnosis based on only cytology and immunohistochemical staining pattern are summarized in Figure 1.

Although a highly specific biomarker for mesothelioma has not yet been demonstrated, a variety of case series reports have been used to identify patterns of positive and negative staining characteristics that are “consistent with” malignant mesothelioma. The primary goal in distinguishing epithelioid types of mesothelioma involves a staining pattern that is generally not seen in adenocarcinomas; this includes positive staining for pankeratin, calretinin, keratin 5/6, and WT1 (but not necessarily all of these), and negative staining for CEA, CD15, Ber-EP4 Moc-31, TTF-1, and B72.3 (Ordonez, 1998; Cappello and Barnes, 2001; Pass et al., 2008). But the heterogeneous histology of mesothelioma (e.g., the involvement of epithelial cells, spindle cells or both cell types) can lead to inconsistent or ambiguous staining patterns that overlap with other tumor types, e.g., certain soft tissue sarcomas (Nicholson et al., 1998; Cappello and Barnes, 2001; Miettinen et al., 2001). In addition, the chromosomal instability of metastatic tumors that often leads to more primitive cell types with indistinct staining patterns may in some cases overlap with those of mesothelioma or soft tissue sarcoma (Morin et al., 2005).

To date, searches for genetic aberrations associated with malignant mesothelioma have failed to identify a highly specific chromosomal abnormality, although several associated lesions based on newer techniques like micro-RNA expression, DNA methylation, telomere status, micronucleus formation, and gene expression profiling are under investigation (Bott et al., 2011; Testa et al., 2011; Jean et al., 2012). In contrast to many other types of solid tumor, mesotheliomas generally lack mutations of the fundamental tumor suppressor genes, p53 and RB. Only a few molecular defects have been identified in mesotheliomas thus far. Pisick and Salgia (2005) reviewed the literature and concluded that while there are several different genetic and signal pathway alterations that can occur in malignant mesothelioma cells and cell lines, these changes are heterogeneous, and therefore not clearly useful for diagnostic purposes at this time. These authors noted that transformation occurs either by direct stimulation of pathways activated by receptor tyrosine kinases or by a loss of genetic material containing tumor suppressor genes that prevent unchecked cell proliferation. The more commonly noted losses occur on the short arms of chromosomes 1 (notably 1p21–p22), 3 (notably 3p21.3), and 9 (notably 9p14/p16), and on the long arms of chromosomes 6 (notably 6q14–q21, 6q16.6–q21, 6q21–q23.2, and 6q25), 15 (notably 15q11.1–q15), and both arms of chromosome 22 (Pisick and Salgia, 2005; Pass et al., 2008). Loss of a copy of chromosome 22 is thought to be the single most consistent karyotypic alteration seen in malignant mesothelioma (Pass et al., 2008). Unfortunately, these patterns of genetic loss also represent genetic variations seen in more common forms of cancer, such as non-Hodgkin's lymphoma, breast, ovarian, prostate, lung and colon cancers (Pisick and Salgia, 2005).

A more recent review on molecular changes observed in mesothelioma by Jean et al. (2012) summarized findings of emerging research on possible genetic markers. These authors identified a slightly wider range of associated chromosomal alterations including losses at 1p, 3p, 4q, 6q, 13p, 14q, and 22q, and gains at 1q, 5p, 7p, 8q, and 17q and noted that more specific mutations on chromosome 22 (e.g., deletions at the neurofibromatosis 2 locus, 22q12), chromosome 9 (e.g., INK4 genes, specifically deletions at cyclin-dependent kinase inhibitor genes CDKN2A and CDKN2B at 9p21.3), and chromosome 17 (TP53 gene deletion at 17p13.1, though less common) have been associated with mesothelioma in recent reports. However, these mutations occur with several other cancer types (Jean et al., 2012). They noted a new association between mesotheliomas and increased microRNA expression of certain types (MiR-31, -141, -192, -193, -200a-c, -203, -205, and -429); greater DNA methylation at certain loci, and increases in markers of telomere length maintenance mechanisms were also reportedly associated with mesotheliomas (Ivanov et al., 2010; Jean et al., 2012). While many of these new associations appear promising as adjuncts to current immunohistochemical staining techniques in discerning mesothelioma from other cancer types, further research is needed to determine their specificity and reliability in refining diagnosis, treatment, and prognosis for mesothelioma.

Other recent observations on mesothelioma genetic markers have focused on mesenchymal membrane receptor tyrosine kinases that dirve downstream cell signaling of cell proliferation, cell cycle control, survival and differentiation (Lemmon and Schlessinger, 2010). First, there is recent evidence suggesting that telomere status and telomere maintenance mechanisms tied to P53, ATRX and DAXX mutations may be helpful in distinguishing more aggressive mesotheliomas (Durant, 2012; Gocha et al., 2013; Tallet et al., 2013). Positive telomerase activity is observed in 91–100% of pleural mesotheliomas in two studies (Dhaene et al., 1998; Au et al., 2011), and telomerase-independent pathways for telomere lengthening through DNA damage repair mechanisms (ALT positive cells) are more commonly observed in sarcomatoid tumors (Heaphy et al., 2011; Durant, 2012; Hu et al., 2012) and persons with multiple endocrine neoplasia type 1 (Gocha et al., 2013). Second, the mutations leading to telomere lengthening and survival of clonally expanding mesothelioma cells using ALT mechanisms are reported to form ALT-associated promyelocytic bodies that may be akin to micronuclei observed in tumor cells and blood polynucleated lymphocytes with exposure to asbestos (Dopp et al., 1995; Bolognesi et al., 2005; Martini et al., 2011). Third, soluble mesothelin-related peptides are overexpressed in persons with mesothelioma (Robinson et al., 2003; Scherpereel et al., 2006) and have been associated with increased micronuclei in peripheral lymphocytes (Martini et al., 2011). And fourth, germline mutations in nuclear deubiquitinase BRCA1-associated protein 1 (BAP1) have been observed in familial clusters of mesothelioma not necessarily linked to asbestos (Testa et al., 2011) and in a fraction of pleural mesotheliomas in other case series (Bott et al., 2011; Jean et al., 2012; Tallet et al., 2013). These more recent associations are all tied into loss of normal cell signaling that occurs in a variety of cancer types, but further investigations may provide more specific genetic markers to help better distinguish mesothelioma diagnosis, treatment, and prognoses in the future (Bott et al., 2011; Jean et al., 2012; Gocha et al., 2013).

Given the preceding issues, it is important to collect and more thoroughly evaluate genetic evidence for mesothelioma cases, particularly in younger cases or those with little or no known amphibole asbestos exposure. Genetic screening in conjunction with assessment of histology and immunohistochemical staining patterns in these cases may result in more definitive identification of the true tissue/cell-type of origin, thereby avoiding misdiagnosis of mesothelioma. This, in turn, would allow for a better identification of tumor-specific risk factors and thereby increase the possibility of a more effective treatment regimen specific for those cancers that are not a true mesothelioma. Screening for genetic aberrations corresponding to tumor types known to mimic mesothelioma would seem to be particularly important in cases where no occupational asbestos exposure has been identified, in cases involving an individual under 50 years old, and in cases with no evidence of pleural plaques, asbestosis, or elevated lung asbestos fiber counts.

**Figure 2:**
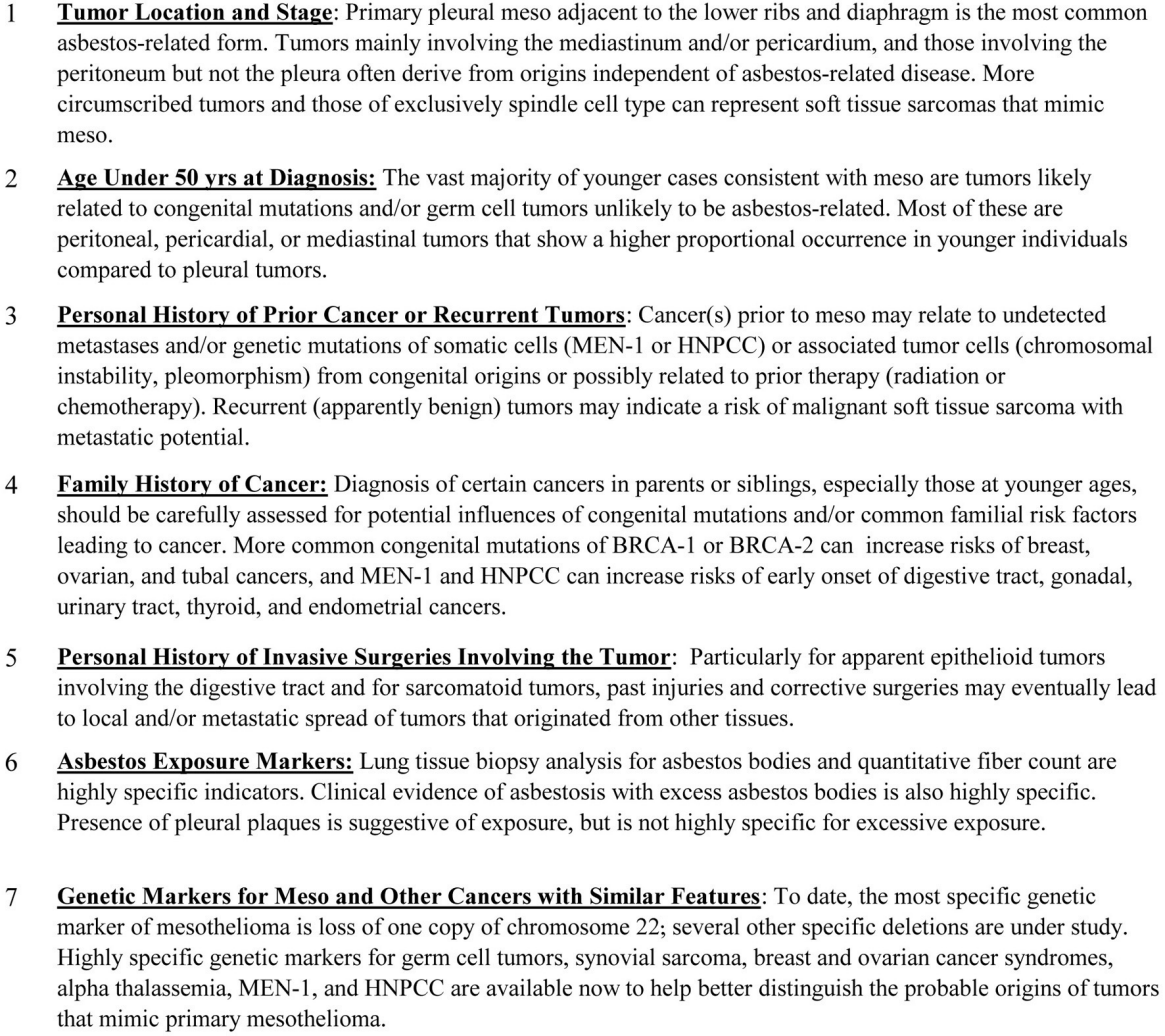
**Additional factors to consider in evaluating suspected asbestos-related mesothelioma**.

Genetic testing of suspected mesothelioma cases with non-distinct staining patterns is apparently an infrequent medical consideration despite the potential value to the patient and to medical science in the form of learning more about the genetics underlying mesothelioma and other tumors types with histological resemblance^1^. Additional factors that can provide important evidence reflecting on the likelihood of misdiagnosis and/or unlikely association with asbestos exposure for tentatively diagnosed mesothelioma cases are provided in Figure 2.

The purpose of this paper is to set out the scientific basis for a registry-based research protocol for the collection of medical information and tissue samples to refine medical knowledge on mesothelioma diagnosis, prognosis, and rates. The basis for genetic testing that may distinguish specific cancer types probably unrelated to asbestos exposure is reviewed, and the utility of a registry-based research program to enhance medical knowledge regarding the etiology of general and specific subtypes of mesothelioma is explored.

## Basis for genetic testing of mesothelioma and histologically similar tumors

### Germ cell tumors and mesothelioma

Advanced stage extragonadal germ cell tumors (GCTs) are known to commonly occur in, or metastasize to, tissues along the body midline from the pineal gland to the sacrum. Due to their multi-potential nature they can exhibit features, both for histology and immunohistochemical markers, of primary pleural or peritoneal malignant mesothelioma. Mesothelioma of the tunica vaginalis is an example of this occurring within the testicle, yet little attention has been given to potential metastatic forms of this tumor and/or to extragonadal GCTs that originate from mutated embryonic cells of the urogenital ridge that may be mistakenly diagnosed as a mesothelioma of the peritoneum or pleura. The occurrence of mesothelioma in children and adolescents without significant or identifiable asbestos exposure (Fraire et al., 1988; Coffin and Dehner, 1992; Niggli et al., 1994; Hubbard, 1997) could plausibly represent a GCT or other congenital/genetic mutations affecting mesenchymal tissues, especially given the much higher background incidence of GCTs relative to that of mesothelioma.

GCTs can arise from germ cell tissues in the testes, the prostate, and other tissue locations (extragonadal GCT) as a result of a mutation and abnormal migration of germ cells during embryonic development (Blossom et al., 1997; Sarma et al., 2006). Blossom et al. (1997) have explained that extragonadal GCTs occur along the body's midline from the presacral areas and retroperitoneum up to the cranium (pineal gland), a pattern that corresponds to the embryologic urogenital ridge extending along the mediastinum and peritoneum from vertebrae C-6 to L-4. Extragonadal GCTs result from malignant transformation of germinal elements that are displaced to extragonadal sites in the absence of any gonadal primary tumor (Blossom et al., 1997). There is no plausible etiologic role of asbestos exposure in the development of extragonadal GCTs.

Blossom et al. (1997) have also noted that approximately 90% of malignant extragonadal GCT occur in men, most of which are diagnosed in the third decade of life or later. While the lifetime probability of developing a GCT (most of which originate from the testes) is only about 0.2% in Caucasian males in the United States, this lifetime risk is far greater than that of mesothelioma. Thus, potential misdiagnosis of these tumors might represent a substantial portion of the tumors mistakenly diagnosed as pleural or peritoneal mesothelioma in males, and may falsely increase the estimated background incidence of mesothelioma. The cause of these tumors is unknown but they are most likely explained by genetic mutation events in early embryonic development (Bosl et al., 1997). Individuals with a history of cryptorchidism are known to exhibit a significantly increased risk of testicular GCT (Bosl et al., 1997; Motzer and Bosl, 2005), as are brothers of individuals with GCTs (Kumar et al., 2005a; Motzer and Bosl, 2005).

The multi-potential nature of GCTs leads to a diverse pathological appearance including combined epithelial and spindle cell presentations that might be mistaken for mesothelioma (Blossom et al., 1997; Bosl et al., 1997; Motzer and Bosl, 2005). Due to this diversity of GCTs, and especially for advanced stage metastatic tumors, common stains and immunohistochemical markers may be unhelpful in identifying the origins of extragonadal GCTs. Fortunately, there are characteristic DNA changes in some tumors affecting chromosome 12 (iso-12p) that may help one to diagnose tumors originating from germ cells from gonadal or extragonadal sites (Bosl et al., 1997; Kumar et al., 2005a; Sarma et al., 2006). Since GCTs are not inhibited in their lines of differentiation, most presentations include two or more histologic patterns (e.g., mixed sarcomatoid and epithelioid as seen with mesothelioma), and males with a history of cryptorchid and dysgenetic testes show familial clustering and a higher frequency of intratubular GCTs (Kumar et al., 2005a).

### Mullerian tissue tumors and mesothelioma

Mullerian tissue tumors, of both breast and ovarian/adnexal origins, include certain mesenchymal subtypes that can spread to either the pleura or peritoneum in females. Breast cancer is a common metastatic tumor affecting the pleura in females (Winston et al., 2000; Kolschmann et al., 2005), and ovarian/adnexal cancers are also reported to spread to both the pleura and the peritoneum at advanced stages (Cormio et al., 2003). Breast cancer risk factors such as BRCA phenotype are now known to carry risks for certain female gonadal cancers that mimic mesothelioma (Hankinson and Danforth, 2006; Cannistra et al., 2008). More specifically, primary papillary serous carcinomas are among the most common forms of aggressive cancer types that originate in female gonadal tissues (i.e., ovarian surface epithelium, fallopian tube epithelium and fimbria, or mullerian tissue gonadal remnants) and can be difficult to distinguish from advanced forms of mesothelioma when the immunohistochemical staining pattern is ambiguous (Kannerstein et al., 1977; Bannatyne and Russell, 1981; Raju et al., 1989; Altaras et al., 1991; Fox, 1993; Alvarado-Cabrero et al., 1997; Crum et al., 2007). The ovarian surface epithelium is comprised of a specialized type of mesothelial cell forming embryonic mesoderm tissue that is a direct descendent of the coelomic epithelium of the embryonic gonadal ridge and of the mullerian duct system (Lauchlan, 1972; Parmley and Woodruff, 1974; Kannerstein et al., 1977; Fox, 1993; Katabuchi and Okamura, 2003; Piek et al., 2004).

Altaras et al. (1991) explained some of the history behind papillary serous carcinomas (PSC) being ultimately distinguished as separate clinical entities from mesotheliomas. Specifically, the peritoneal location of PSC is explained by the common coelomic ancestry of the mullerian duct epithelium and the peritoneal mesothelium. Mullerian differentiation can occur in a variety of metaplastic and neoplastic ways in diverse tissue locations, such as PSC, ovarian tumors of common epithelial origin, endometriosis, endosalpingiosis, and epithelial inclusions of the ovary and lymph nodes (Altaras et al., 1991). Diffuse PSC of the peritoneum without overt involvement of the ovary was initially described as malignant mesothelioma, until Kannerstein et al. (1977) identified histological criteria that distinguished PSC from mesotheliomas and from all extraovarian epithelial tumors. However, PSC of peritoneal and ovarian origin remain indistinguishable from each other by histopathology (Altaras et al., 1991).

More advanced-stage tumors and metastases from mullerian cells may become more primitive (undifferentiated) such that the tumor cell histology and immunohistochemical markers of the ovarian surface epithelial cells cannot be readily distinguished from less specialized mesothelial tumors that may originate in the peritoneal lining tissue (Hernandez et al., 1984; August et al., 1985; Cramer et al., 1987; Lund et al., 1991; Sakamoto et al., 1994; Gilks et al., 2008).

Some key tumor immunohistochemical markers (e.g., positive calretinin, CK7, and WT-1, with negative CEA) and cellular characteristics (epithelioid, poorly differentiated) are commonly seen for both mesothelioma and PSC (Fox, 1993; Schorge et al., 2000; Shimizu et al., 2000; Al-Hussaini et al., 2004; McCoubrey et al., 2005; Kobel et al., 2008). Indeed, PSC in many ways can mimic epithelioid mesothelioma, but unlike mesothelioma, PSC is linked to hereditary and sporadic mutations of two breast cancer susceptibility genes (BRCA-1 and BRCA-2). Primary epithelial cancers of the fallopian tubes (particularly the fimbria) appear to be linked to more aggressive and early-onset PSC in women with the BRCA-1 mutation (Sobol et al., 2000; Zweemer et al., 2000; Aziz et al., 2001; Paley et al., 2001; Levine et al., 2003; Piek et al., 2003; Finch et al., 2006; Medeiros et al., 2006; Kindelberger et al., 2007), while ovarian surface epithelium tumors have been linked to both BRCA-1 and BRCA-2 mutations (Werness et al., 2000; Colgan et al., 2001, 2002; Piura et al., 2001; Risch et al., 2001; Agoff et al., 2002; Brose et al., 2002; Geisler et al., 2002; Hilton et al., 2002; Cass et al., 2005). More advanced PSC tumors originating from ovarian surface epithelium or tubal fimbria epithelium are subject to genomic instability, loss of WT-1 gene expression, and the presence of both BRCA and p53 mutations, sometimes making the site or origin difficult to distinguish in more advanced stages of the disease (Schorge et al., 2000; Piek et al., 2004; Kindelberger et al., 2007; Lee et al., 2007; Gilks et al., 2008).

Since PSC can originate as an occult mullerian neoplasm that commonly proliferates at other peritoneal sites (Colgan et al., 2001, 2002; Cass et al., 2005; Powell et al., 2005; Finch et al., 2006; Chand et al., 2007), the pathological (histology and staining) evaluation of PSC often does not identify the original tumor site and should be augmented with detailed analysis of the patient's clinical and family history and BRCA phenotype in order to assign the final diagnosis with reasonable medical certainty (Risch et al., 2001; Piek et al., 2004; Kindelberger et al., 2007; Gilks et al., 2008). BRCA-positive families are noted to have higher cancer rates affecting the colon, prostate, pancreas and peritoneum, as well as melanoma (Struewing et al., 1997; Frank, 2001; Paley et al., 2001; Al-Hussaini et al., 2004). Three familial genetic syndromes associated with excess ovarian cancer risk have been identified: certain BRCA-1 or BRCA-2 mutations result in Breast Ovarian Cancer Syndrome, Site-Specific Ovarian Cancer Syndrome, and Hereditary Non-Polyposis Colon Cancer (or Lynch II Syndrome) which are associated with mutations in DNA mismatch repair genes in affected families (Muto, 2005).

In contrast, PSC has not been specifically associated with asbestos exposure (Fox, 1993), and peritoneal mesothelioma are predominantly associated with relatively high exposures to primarily amphibole forms of asbestos (Sporn and Roggli, 2004; Bofetta and Stayner, 2006; Yarborough, 2007; Gibbs and Berry, 2008).

### Congenital heart disease and pericardial mesothelioma

Pericardial mesothelioma is estimated to comprise only 0.7–1% of all mesotheliomas (Papi et al., 2005; Molina Garrido et al., 2006) and lacks a specific immunohistochemical staining pattern that would distinguish this lesion from mesotheliomas originating at other sites (Reis-Filho et al., 2002; Papi et al., 2005). In contrast to pleural mesothelioma, the association of asbestos exposure with pericardial mesothelioma is unclear (Eren and Akar, 2002; Val-Bernal et al., 2002; Lagrotteria et al., 2005; Papi et al., 2005; Luk et al., 2008). Although a few reported cases have occurred in individuals with occupational asbestos exposure (Kahn et al., 1980; Beck et al., 1982; Thomason et al., 1994; Fujiwara et al., 2005), it appears that the vast majority of recently reported cases had no known asbestos exposure (Quinn et al., 2000; Hirano et al., 2002; Reis-Filho et al., 2002; Val-Bernal et al., 2002; Susman et al., 2004; Yakirevich et al., 2004; Erdogan et al., 2005; Lagrotteria et al., 2005; Maruyama et al., 2006; Molina Garrido et al., 2006; Doval et al., 2007; Vornicu et al., 2007; Akamoto et al., 2008; Santos et al., 2008), and many were reported in younger individuals (e.g., age 19–50 years). Malignant mesothelioma of the pleural cavity was also reported to occur in a 15-year old boy with rheumatic heart disease and a prosthetic aortic valve implant; this child was reported to have no history of asbestos or chemical exposure (Tewari et al., 1989).

As reviewed by Kumar et al. (2005b), the heart is one of the first organs to form and function during early embryonic development. Developmental errors in mesenchymal tissue migration are just one example of genetic errors leading to certain types of congenital heart disease, e.g., involving various anomalies of the outflow tract, some due to failure of fusion and others due to failure of septation. Parallel to the genesis of some extragonadal GCTs from abnormal germ cell migration along the embryological urogenital ridge, some congenital heart defects may be caused by abnormal development of neural crest-derived cells, whose migration into the heart is required for proper formation of the outflow tracts. Many congenital heart defects are related to trisomies of chromosomes 13, 15, 18, and 21, and deletions in chromosome 22 (22q11.2) are thought to play a major role in congenital heart disorders of the conotruncus and the brachial arches (Kumar et al., 2005b). Accordingly, it seems appropriate that identification of a case of malignant mesothelioma involving congenital heart disease would initiate genetic screening to help define the common congenital defects that may relate to the onset of both diseases in the same individual. Further research is also recommended to examine the incidence of malignant mesothelioma among individuals with various forms and severity of congenital heart disease, and perhaps also in relation to rheumatic heart disease.

### Synovial sarcomas presenting as mesothelioma

There is no demonstrated association between synovial sarcoma and asbestos exposure, although synovial sarcoma of the pleura is difficult to distinguish from sarcomatoid mesothelioma without the use of genetic screening (Nicholson et al., 1998; Cappello and Barnes, 2001; Colwell et al., 2002; Weinbreck et al., 2007). These tumors are thought to originate from primitive pluripotent mesenchyme cells capable of sarcomatous differentiation. This tumor can be highly aggressive, involving metastases to distant locations (Siegel et al., 2007; Eilber and Dry, 2008), and consistent with this they have been observed to occur in tissues with little or no associated synovium. Indeed, synovial sarcoma does not arise from or differentiate toward synovium—it is an unfortunate misnomer since the vast majority arise in deep soft tissue of extremities, especially around the knee. Because of this it has been proposed that synovial sarcoma be renamed as carcinosarcoma or spindle cell carcinoma of soft tissue due to its immunoreactivity to cytokeratins and EMA. Relevant to mistaken pleural mesotheliomas, synovial sarcomas have been prominently noted to occur in the pleural cavity (Gaertner et al., 1996; Nicholson et al., 1998; Caliandro et al., 2000; Cappello and Barnes, 2001; Colwell et al., 2002; Vohra et al., 2004; Lee et al., 2006; Tsukioka et al., 2006; Galetta et al., 2007; Iwata et al., 2007; Mukhopadhyay and Aubry, 2007; Satoh et al., 2007; Weinbreck et al., 2007), as well as in the lung (Hosono et al., 2005; Taylor et al., 2005). Other reported sites include the peritoneal cavity (Vera et al., 2006; Gofman et al., 2007), and the stomach (Makhlouf et al., 2008).

Synovial sarcoma is a relatively rare malignancy that typically occurs in adolescents and young adults between the ages of 15 and 50 years of age and most commonly affects the extremities in the vicinity of large joints such as the knee or the thigh (Cadman et al., 1965; Cordon-Cardo, 1997; Nicholson et al., 1998; Cappello and Barnes, 2001; Kumar et al., 2005c) and the hands or feet (Michal et al., 2006). Because synovial sarcoma can be difficult to distinguish from reactive mesothelial proliferation and sarcomatoid mesothelioma by use of histology and immunohistochemical markers alone (Shiraki et al., 1989; Moran et al., 1992; Nicholson et al., 1998; Miettinen et al., 2001; Carbone et al., 2002; Gladish et al., 2002; Vohra et al., 2004; Taylor et al., 2005; Michal et al., 2006; Rdzanek et al., 2006), it represents another alternative carcinoma to consider when evaluating suspected sarcomatoid mesothelioma cases. This has been particularly true since the discovery of highly specific genetic lesions [t(x;18), SYT-SSX1, SSX2, and SSX4 fusion genes] in synovial sarcoma tumors that clinically distinguish this particular this tumor type (Colwell et al., 2002; Amary et al., 2007; Weinbreck et al., 2007). Indeed, Sandberg (2002) provides a summary of specific chromosomal translocations corresponding to a variety of soft tissue sarcomas including synovial sarcoma where—*the translocation is the sole cytogenetic anomaly, indicating the probable causative role of this translocation in the genesis of these tumors.* In synovial sarcoma, the normal SYT gene encodes a transcription factor whereas the SSX1 and SSX2 genes produce proteins that are transcription inhibitors. The available evidence suggests that the specific type of translocation may be related to the aggressiveness of the synovial sarcoma and its prognosis. The most common sites of metastases are the lung/mediastinum, the skeleton, and regional lymph nodes (Hosono et al., 2005; Kumar et al., 2005c; Michal et al., 2006).

Two additional soft tissue sarcomas should be mentioned here because they have the potential to occur as poorly differentiated metastatic tumors with staining characteristics that are difficult to distinguish from sarcomatoid mesothelioma, but they can be positively identified by their unusual chromosomal alterations (Kumar et al., 2005a). Desmoplastic small round cell tumor, a tumor of unknown hisotogenesis in the peritoneum, shows a specific translocation [t(11;22)(p13;q12)], involving the EWS-WT-1 gene fusion product in 90% of cases. Endometrial stromal sarcoma, a tumor that may occur as distant metastases decades after hysterectomy, also shows a specific translocation [t(7;17)(p15;q21)], involving JAZF1-JJAZ1 gene fusion product in about 65% of cases. These tumors are relatively rare sarcomas that have sufficiently unique markers to distinguish the origin of poorly differentiated spindle cell tumors that may be mistaken as mesotheliomas.

### Inherited cancer susceptibility syndromes and mesothelioma

Certain inherited disorders produce multiple cancers that are prone to develop chromosomal instability and present at a late stage with histological features that mimic disseminated mesothelioma (Hawley and Pandolfi, 2008). Two examples of such inherited disorders are Multiple Endocrine Neoplasia Syndrome-1 (MEN-1) and Hereditary Non-Polyposis Colon Cancer (HNPCC). Both syndromes are known to have diagnostic genetic mutations in somatic cells that identify the syndrome and overt clinical outcome which consists of multiple, but often survivable, cancers in affected subjects and their blood relatives.

MEN-1 involves a germline mutation at the 11q13 (a tumor suppressor gene) which encodes for a protein menin and is associated with increased risk of parathyroid, endocrine (thyroid, prostate, testicular, breast, and ovarian), pancreas, and pituitary neoplasia (mostly adenomas) (Mayer, 2005; Morin et al., 2005). MEN-1 is known to occur in conjunction with Zollinger-Ellison Syndrome (ZES) in both sporadic and heritable forms, with an estimated 50% penetration among blood relatives. ZES leads to the formation of gastrinomas in the pancreas and/or duodenum that lead to hypergastrinemia and intractable peptic ulcer disease, however the most common and earliest manifestation is usually primary hyperparathyroidism. Clinical markers for ZES are elevated fasting gastrin (>150 pg/ml) and sometimes hyperparathyroidism resulting in elevated blood calcium, parathyroid hyperplasia, and other sequelae (Goyal, 2005). The late stages of MEN-1 are associated with increasing chromosomal instability among tumors that may have metastasized to other tissues decades earlier, or involving proliferation from surgical scars of the stomach, since the only way to resolve duodenal hypergastrinemia is with distal gastrectomy. Late stage tumors of MEN-1 patients have been associated with chromosomal instability that may plausibly lead to local tissue invasion and conversion to mixed neoplastic cell types including soft tissue sarcomas that may mimic mesotheliomas. Thus, it is suggested that persons with characteristic prior cancers and/or ZES and distal gastrectomy who present with apparent mesothelioma should be tested for the somatic cell mutation (11q13) of the MEN-1 tumor suppressor gene, in addition to testing for other translocations that may be diagnostic for various types of soft tissue sarcoma that may be relevant to the tumor/site (Sandberg, 2002).

Similarly, HNPCC involves germline mutations at certain loci (2p16, 3p21.3, and 7p22) that are associated with the formation of carcinoid tumors of the duodenum and ascending colon (Mayer, 2005; Morin et al., 2005; Hawley and Pandolfi, 2008). As with MEN-1, this germline mutation is thought to have a high penetration rate, and often involves multiple additional tumor sites including endometrial, ovarian, stomach, small bowel, and ureter carcinoma. Also, late stage tumors in HNPCC patients are associated with chromosomal instability that may plausibly lead to local tissue invasion and conversion to mixed neoplastic cell types including soft tissue sarcomas that may mimic mesotheliomas. Thus, persons with characteristic prior cancers who present with apparent mesothelioma should be tested for the somatic cell mutations of HNPCC, in addition to testing for other translocations that may be diagnostic for various types of soft tissue sarcoma that may be relevant to the tumor/site (Sandberg, 2002).

## Discussion

Based on the preceding research summary, a preliminary genetic screening matrix has been outlined in Table 1 that could be used as a starting point for further characterizing and understanding the clinical and genetic features of malignant mesothelioma and other primary cancers that may sometimes mimic asbestos-related mesothelioma. As noted in the Introduction, there are many additional candidate features that could be added to Table 1 as the associations between mesothelioma and various genetic markers are further clarified. This preliminary matrix could assist in developing future research plans and incorporating other clinical observations that may help to fulfill the goal of obtaining unambiguous diagnostic criteria for various forms of malignant mesothelioma and tumors that may mimic mesothelioma.

**Table 1:** **Suggested matrix for genetic screening of suspected malignant mesothelioma cases**.

**Patient type**	**Specifc genetic lesions**	**Rationale and possibly associated clinical eatures**
All Patients	Deletions in tumor tissue DNA at 1p21-p22, 3p21.3, 9p14/p16, 6q14-q21, 6q16.6-q21, 6q21-q23.2, 6q25, 15q11.1-q15; and loss of a copy of chromosome 22	Most commonly associated genetic lesions identified in mesothelioma but overlapping with other cancer types. Monitor statistical associations to identify which specific deletions are most specific and diagnostic for general and site-specific mesothelioma
Patients with prominent epithelioid histology and primary midline or mediastinal tumor occurrence	Germ cell tumor markers in tumor tissue: iso12p, often multiple copies; widespread gene loss across most chromosome arms, and non-random gains in chromosomes 1, 7, 12, 21, 22, and X	Germ cell tumors can metastasize widely, and extragonadal germ cell tumors are known to occur in nodes and tissues along the embryonic urogenital ridge from the cranium to the presacral region (C6 to L4) due to abnormal germ cell migration. Treatment and prognosis may be very different compared to primary mesothelioma. Clinical correlates may include history of cryptorchidism and family history of testicular cancer or male sibling leukemia or lymphoma
Patients with epithelioid histology and primary peritoneal tumor occurrence and a family history or personal history of breast and/or ovarian cancer	Testing of tumor tissue and somatic cells for mutations in BRCA-1 and BRCA-2 for meso with history of ovarian or breast cancer; mutations at 2p16, 3p21.3, 7p22 for history of multiple endocrine cancer; and del(22q11.2) and trisomy 13, 15, 18 or 21 and for peritoneal meso with heart valve defects	Serous epithelioid cancers from mullerian tissues can present as peritoneal metastases from occult tumors of mullerian tissues (e.g., ovarrian surface epithelium, fallopian tubes and fimbria) and are difficult to distinguish from mesothelioma without thorough pathological evaluation. Serous carcinomas may occur sporadically or with familial syndromes including Breast Ovarian Cancer Syndrome, Site Specific Ovarian Cancer Syndrome, and Hereditary Non-Polyposis Colon Cancer
Patients with prominent spindle cell histology and possible history of severe synovial trauma and/or recurrent synovial growths	Synovial sarcoma markers in tumor tissue: translocation (x;18) leading to SSX1 or SSX2 fusion gene transcripts	Synovial sarcomas generate from mutation of mesenchymal tissues, can metastasize to locations mimicking true mesothelioma, and can have widely varied clinical presentation and prognosis. Genetic tracking of these tumors may assist to better characterize primary site, treatment and prognosis. Recurrent synovial or ganglionic cysts, and severe or repeated trauma to synovial tissues may reflect higher risks
Patients with personal and/or family history of certain additional primary cancers (e.g., thyroid, prostate, testes, breast, ovary, pancreas, GI tract, ureter, endometrium)	Somatic cell mutations: 11q13 for history of possible multiple endocrine neoplasia/ Zollinger-Ellison Syndrome, MEN-1/ZES; and 2p16, 3p21.3, 7p22 mutations for history of possible hereditary non-polyposis colon cancer, HNPCC	In advanced stages of invasive/metastatic cancers from MEN-1 or HNPCC, chromosomal instability may lead to histopathologic features that mimick mesothelioma. Genetic tracking may assist to better characterize primary site, treatment and prognosis. MEN-1/ZES includes diagnostic gastrinomas of duodenum/pancrease with intractable peptic ulcer disease often requiring gastrectomy. HNPCC includes diagnostic carcinoid tumors of duodenum and ascending colon

Diagnosing a tumor as mesothelioma based on histologic and immunohistochemical staining characteristics alone can lump together several mesenchymal tumor types of different origins that should be treated as distinct clinical entities with unique risk factors, prognosis, and best treatment modalities. Several mesenchymal tumors that are difficult to distinguish based on histology and staining characteristics are already known to be distinguished by genetic markers in somatic or tumor cells.

The number of mullerian or germ cell tumors, advanced metastatic tumors, or mistaken synovial sarcomas that may have no plausible connection to asbestos exposure will remain obscure without appropriate research that supports a more definitive, evidence- based decision matrix for correct diagnosis. While the prognosis of any given individual's cancer can be difficult to predict, this research may identify genetic and/or clinical characteristics that presently, or in the future, will aid in more accurate prognostic predictions and perhaps more tumor-specific and efficacious treatments. For example, some forms of synovial sarcoma that remain circumscribed and relatively indolent are survivable without the aggressive clinical interventions typical of the treatment of malignant mesothelioma. Similarly, the confirmation of germ cell tumor origins would call for more focused clinical investigations and interventions that greatly differ from mesothelioma treatments. By adopting better diagnostic criteria for those mesenchymal tumor subtypes that may be mistaken as mesothelioma, physicians will improve epidemiologic estimates of the incidence and prevalence of the tumor types discussed above. Thus, this approach would ultimately improve dose-response relationships in the low dose region for asbestos and mesothelioma as well as that of other apparent risk factors for this deadly disease.

As discussed earlier, the proportion of future mesothelioma cases that can be credibly attributed to asbestos is likely to lessen because the highly potent amphibole exposures were largely curtailed in the 1960s and the typical latency period of 20–40 years since first exposure has now transpired. The incidence of pleural mesotheliomas that are more specifically associated with asbestos is projected to continue declining, while peritoneal mesothelioma incidence (associated primarily with non-asbestos causes) has been essentially flat for decades (Teta et al., 2008; Moolgavkar et al., 2009). Parallel to mesothelioma risk trends, asbestosis is a fibrotic interstitial lung disease with a comparable latency period that has also generally diminished in both incidence and clinical severity in the past two decades. This trend has raised questions about how many valid asbestosis cases arise from the many claims that today are often based solely on equivocal radiographic findings and questionable occupational history of asbestos exposure (Bang et al., 2008; Mizell et al., 2009; Harding and Darnton, 2010).

Physicians are urged to recognize that the proportion of mesothelioma cases unrelated to asbestos will likely increase in coming decades, and that better vigilance will be needed for proper diagnosis, treatment, and prevention. This will undoubtedly involve the development and use of more definitive diagnostic tools like genetic screening that can help differentiate asbestos-related mesothelioma from other mesenchymal tumor subtypes or advanced metastatic tumors that can mimic the histopathological presentation of mesothelioma.

A national or international registry-based mesothelioma research program may be an achievable and appropriate means for augmenting the knowledge base on clinical features and genetic markers for distinguishing specific forms of mesothelioma and tumors that may mimic mesothelioma. Since the annual number of mesothelioma cases is relatively small, the scope of research each year may be reasonably defined. Incident case detection could be linked to existing state and/or national cancer registries, with the registry research program overseen by a steering committee with outside peer review resources. A working group could be tasked with developing appropriate research protocols for collection of needed clinical and family history information in addition to samples of tumor tissue and somatic cells for genetic screening. The scope of analysis and quality control procedures of the working group should be clearly defined and transparent, with assurance of evidence-based data analysis and outside peer review of findings in a manner that also assures objectivity and patient privacy.

In conclusion, the identification of relatively specific clinical features and genetic markers that may avoid misdiagnosis of mesothelioma and other mesenchymal tumor subtypes or advanced metastatic tumors is an important future research need. Currently, the diagnosis of mesothelioma rests largely on histology and staining patterns that are sometimes inconclusive. It is recommended that further research be directed at identifying those genetic and clinical features unique to malignant mesothelioma and the cancers that may mimic its histopathology. This research could be fostered through a national or international mesothelioma registry with requisite medical history questionnaire and tumor/somatic tissue submission, hopefully leading to more specific diagnostic tools, better disease classification and incidence data, and a uniform and enhanced database for understanding the natural history and prognosis of various mesothelioma subtypes.

### Conflict of interest statement

The authors are research scientists employed by scientific consulting firms that are paid to conduct research for private clients relating to questions raised in regulatory and legal arenas. Each of the authors has conducted such research on behalf of clients with alleged asbestos liabilities, including expert witness activities. This work was funded solely by the authors and their institutions, without financial or technical assistance from any client. The authors declare that the research was conducted in the absence of any commercial or financial relationships that could be construed as a potential conflict of interest.

## Footnotes

^1^Each of the research categories proposed in this manuscript is based on a compilation of notes from actual mesothelioma claims with findings that supported the alternative diagnosis. However, current privacy laws in the United States prevent reporting specific details of these cases here. Establishing a registry-based research program as proposed in this paper would generate the epidemiologic and clinical information for the improvement of diagnosis and identification of risk factors for this cancer in a manner that protects patient privacy. A registry would provide a neutral setting outside of the litigation arena where legal maneuvering would not hamper necessary data collection and analysis. Unfortunately, it has been the experience of one of the authors (BDK) that information that could improve disease causation analysis for mesothelioma cases has been blocked when a legal case has been filed. These attorneys may not realize that a more definitive diagnosis might lead to a more effective treatment and a better prognosis for the affected individual.

## References

[B1] Agoff S. N. Mendelin J. E. Grieco V. S. Garcia R. L. (2002). Unexpected gynecologic neoplasms in patients with proven or suspected BRCA-1 or -2 mutations: implications for gross examination, cytology, and clinical follow-up. Am. J. Surg. Pathol. 26, 171–178 10.1097/00000478-200202000-00003 11812938

[B2] Akamoto S. Ono Y. Ota K. Suzaki N. Sasaki A. Matsuo Y. (2008). Localized malignant mesothelioma in the middle madiastinum. Surg. Today 38, 635–638 10.1007/s00595-007-3679-1 18612789

[B3] Al-Hussaini M. Stockman A. Foster H. McCluggage W. G. (2004). WT-1 assists in distinguishing ovarian from uterine serous carcinoma and in distinguishing between serous and endometrioid ovarian carcinoma. Histopathol 44, 109–115 10.1111/j.1365-2559.2004.01787.x 14764054

[B4] Altaras M. M. Aviram R. Cohen I. Cordoba M. Weiss E. Beyth Y. (1991). Primary peritoneal papillary serous adenocarcinoma: clinical and management aspects. Gynecol. Oncol. 40, 230–236 10.1016/0090-8258(90)90283-Q 2013445

[B5] Alvarado-Cabrero I. Navani S. S. Young R. H. Scully R. E. (1997). Tumors of the fimbriated end of the fallopian tube: a clinicopathologic analysis of 20 cases, including nine carcinomas. Int. J. Gynecol. Pathol. 16, 189–196 10.1097/00004347-199707000-00001 9421082

[B6] Amary M. F. Berisha F. Bernardi Fdel C. Herbert A. James M. Reis-Filho J. S. (2007). Detection of SS18-SSX fusion transcripts in formalin-fixed paraffin-embedded neoplasms: analysis of conventional RT-PCR, qRT-PCR and dual color FISH as diagnostic tools for synovial sarcoma. Mod. Pathol. 20, 482–496 10.1038/modpathol.3800761 17334349

[B7] Au A. Y. M. Hackl T. Yeager T. R. Cohen S. B. Pass H. I. Harris C. C. (2011). Telomerase activity in pleural malignant mesotheliomas. Lung Cancer 73, 283–288 10.1016/j.lungcan.2010.12.023 21277646 PMC3135747

[B8] August C. Z. Murad T. M. Newton M. (1985). Multiple focal extraovarian serous carcinoma. Int. J. Gynecol. Pathol. 4, 11–23 10.1097/00004347-198501000-00002 3880151

[B9] Aziz S. Kuperstein G. Rosen B. Cole D. Nedelcu R. McLaughlin J. (2001). A genetic epidemiological study of carcinoma of the fallopian tube. Gynecol. Oncol. 80, 341–345 10.1006/gyno.2000.6095 11263928

[B10] Bang K. M. Mazurek J. M. Syamlal G. Wood J. M. (2008). Asbestosis mortality surveillance in the United States, 1970–2004. Int. J. Occup. Environ. Health 14, 161–169 10.1179/oeh.2008.14.3.161 18686715

[B11] Bannatyne P. Russell P. (1981). Early adenocarcinoma of the fallopian tubes. Diag. Gynecol. Obstet. 3, 49–60 10.1007/s00595-007-3679-1 7215124

[B12] Beck B. Konetzke G. Ludwig V. Rothig W. Sturm W. (1982). Malignant pericardial mesotheliomas and asbestos exposure: a case report. Am. J. Ind. Med. 3, 149–159 10.1002/ajim.4700030205 7137171

[B13] Berman D. W. Crump K. S. (2003). Final Draft: Technical Support Document for a Protocol To Assess Asbestos-Related Risk. Washington, DC: Office of Solid Waste and Emergency Response, U.S. Environmental Protection Agency

[B14] Berman D. W. Crump K. S. (2008a). A meta-analysis of asbestos-related cancer risk that addresses fiber size and mineral type. Crit. Rev. Toxicol. 38, 49–73 10.1080/10408440802273156 18686078

[B15] Berman D. W. Crump K. S. (2008b). Update of potency factors for asbestos-related lung cancer and mesothelioma. Crit. Rev. Toxicol. 38, 1–47 10.1080/10408440802276167 18671157

[B169] Bernstein D. M. Rogers R. A. Sepulveda R. Donladson K. Schuler D. Gaering S. (2010). The pathological response and fate in the lung and pleura of chrysotile in combination with fine particles compared to amosite asbestos following short-term inhalation exposure: interim results. Inhal. Toxicol. 22, 937–962 10.3109/08958378.2010.497818 20695727

[B170] Bernstein D. M. Rogers R. A. Sepulveda R. Donaldson K. Schuler D. Gaering S. (2011). Quantification of the pathological response and fate in the lung and pleura of chrysotile in combination with fine particles compared to amosite-asbestos following short-term inhalation exposure. Inhal. Toxicol. 23, 372–391 10.3109/08958378.2011.575413 21639707 PMC3128827

[B168] Bernstein D. M. Rogers R. Smith P. (2005). The biopersistence of Canadian chrysotile asbestos following inhalation: final results through 1 year after cessation of exposure. Inhal. Toxicol. 17, 1–14 10.1080/08958370590885663 15764479

[B16] Blossom G. B. Steiger Z. Stephenson L. W. (1997). Neoplasms of the mediastinum, in Cancer, Principles and Practice of Oncology, 5th Edn., eds. DeVita V. T. Hellman S. Rosenberg S. A. R (Philadelphia, PA: Lippincott-Raven), 951–969

[B17] Bofetta P. (2007). Epidemiology of peritoneal mesothelioma: a review. Ann. Oncol. 18, 985–990 10.1093/annonc/mdl345 17030547

[B18] Bofetta P. Stayner L. T. (2006). Pleural and peritoneal neoplasms, in Cancer Epidemiology and Prevention, 3rd Edn., eds Schottenfeld D. Fraumeni J. F. Jr (London: Oxford University Press), 659–673 10.1093/acprof:oso/9780195149616.003.0034

[B18a] Bolognesi C. Martini F. Tognon M. Filiberti R. Neri M. Perrone E. (2005). A moleculr epidemiology case control study on pleural malignant mesothelioma. Cancer Epidemiol. Biomark. Prev. 14, 1741–1746 10.1158/1055-9965.EPI-04-0903 16030111

[B19] Bosl G. J. Sheinfeld J. Bajorin D. F. Motzer R. J. (1997). Cancer of the testis, in Cancer: Principles and Practice of Oncology, 5th Edn., eds DeVita V. T. Hellman S. Rosenberg S. A. (Philadelphia: Lippincott-Raven), 1397–1425

[B20] Bott M. Brevet M. Taylor B. S. Shimizu S. Ito T. Wang L. (2011). The nuclear deubiquitinase BAP1 is commonly inactivated by somatic mutations and 3p21.1 losses in malignant pleural mesothelioma. Nat. Genet 43, 668–672 10.1038/ng.855 21642991 PMC4643098

[B21] Brose M. S. Rebbeck T. R. Calzone K. A. Stopfer J. E. Nathanson K. L. Weber B. L. (2002). Cancer risk estimates for BRCA1 mutation carriers identified in a risk evaluation program. J. Natl. Cancer Inst. 94, 1365–1372 10.1093/jnci/94.18.1365 12237282

[B22] Burdorf A. Jarvholm B. Siesling S. (2007). Asbestos exposure and differences in occurrence of peritoneal mesothelioma in the Netherlands and Sweden. Occup. Environ. Med. 64, 839–842 10.1136/oem.2006.031724 17567726 PMC2095382

[B23] Cadman M. L. Soule E. H. Kelly P. J. (1965). Synovial sarcoma: an analysis of 134 tumors. Cancer 18, 613–627 14278894 10.1002/1097-0142(196505)18:5<613::aid-cncr2820180510>3.0.co;2-v

[B24] Caliandro R. Terrier P. Regnard J. F. De Montpréville V. Ruffié P. (2000). Primary biphasic synovial sarcoma of the pleura. Rev. Mal. Respir. 17, 498–502 10859770

[B25] Cannistra S. A. Gershenson D. M. Recht A. (2008). Ovarian cancer, fallopian tube carcinoma, and peritoneal carcinoma, in Cancer: Principles and Practice of Oncology, 8th Edn, eds DeVita V. T. Lawrence T. S. Rosenberg Lippincott S. A. (Philadelphia: Williams and Wilkins), 1568–1594

[B26] Cappello F. Barnes L. (2001). Synovial sarcoma and malignant mesothelioma of the pleura: review, differential diagnosis and possible role of apoptosis. Pathology 33, 142–148 10.1080/00313020120038728 11358044

[B27] Carbone M. Rizzo P. Powers A. Bocchetta M. Fresco R. Krausz T. (2002). Molecular analyses, morphology and immunohistochemistry together differentiate pleural synovial sarcomas from mesotheliomas: clinical implications. Anticancer Res. 22, 3443–3448 12552937

[B28] Cass I. Holschneider C. Datta N. Barbuto D. Walts A. E. Karlan B. Y. (2005). BRCA- mutation—associated fallopian tube carcinoma: a distinct clinical phenotype? Obstet. Gynecol. 106, 1327–1334 10.1097/01.AOG.0000187892.78392.3f 16319259

[B29] Chand M. Moore P. J. Clarke A. D. Nash G. F. Hickisk T. (2007). A diagnostic dilemma following risk-reducing surgery for BRCA1 mutation—a case report of primary papillary serous carcinoma presenting as sigmoid cancer. World J. Surg. Oncol. 5, 102 10.1186/1477-7819-5-102 17850658 PMC2075500

[B171] Churg A. (1998). Nonneoplastic diseases caused by asbestos, in Pathology of Occupational Lung Disease, 2nd Edn, eds Churg A. Green F. H. Y. (Baltimore: Williams and Wilkins), 277–339

[B30] Coffin C. M. Dehner L. P. (1992). Mesothelial and related neoplasms in children and adolescents: a clinicopathologic and immunohistochemical analysis of eight cases. Pediatr. Pathol. 12, 333–347 10.3109/15513819209023314 1384016

[B31] Colgan T. J. Boerner S. L. Murphy J. Cole D. E. Narod S. Rosen B. (2002). Peritoneal lavage cytology: an assessment of its value during prophylactic oophorectomy. Gynecol. Oncol. 85, 397–403 10.1006/gyno.2002.6638 12051865

[B32] Colgan T. J. Murphy J. Cole D. E. Narod S. Rosen B. (2001). Occult carcinoma in Prophylactic Oophorectomy specimens: prevalence and association with BRCA germline mutation status. Am. J. Surg. Pathol. 25, 1283–1289 10.1097/00000478-200110000-00009 11688463

[B33] Colwell A. S. O'Cunha J. Vargas S. O. Parker B. Dal Cin P. Maddaus M. A. (2002). Synovial sarcoma of the pleura: a clinical and pathologic study of three cases. J. Thoracic Cardiovasc. Surg. 124, 828–832 10.1067/mtc.2002.124242 12324743

[B34] Cordon-Cardo C. (1997). Molecular biology of sarcomas, In: Cancer, principles and practice of oncology, 7th Edn., eds DeVita V. T. Hellman S. Rosenberg S. A. (Philadelphia, PA: Lippincott-Raven), 1731–1737

[B35] Cormio G. Rossi C. Cazzolla A. Resta L. Loverro G. Greco P. (2003). Distant metastases in ovarian carcinoma. Int. J. Gynecol. Cancer 13, 125–129 10.1046/j.1525-1438.2003.13054.x 12657111

[B36] Cramer S. F. Roth L. M. Ulbright T. M. Mazur M. T. Nunez C. A. Gersell D. J. (1987). Evaluation of the reproducibility of the World Health Organization classification of common ovarian cancers. Arch. Pathol. Lab. Med. 111, 819–829 3632299

[B37] Crum C. P. Drapkin R. Kindelberger D. Medeiros F. Miron A. Lee Y. (2007). Lessons from BRCA: the tubal fimbria emerges as an origin for pelvic serous cancer. Clin. Med. Res. 5, 35–44 10.3121/cmr.2007.702 17456833 PMC1855333

[B38] Dhaene K. Hubner R. Kumar-Singh S. Weyn B. Van Marck E. (1998). Telomerase activity in human pleural mesothelioma. Thorax 53, 195–918 10.1136/thx.53.11.915 10193387 PMC1745102

[B39] Dopp E. Saedler J. Stopper H. Weiss D. G. Schiffmann D. (1995). Mitotic disturbances and micronucleus induction in Syrian hamster embryo fibroblast cells caused by asbestos fibers. Environ. Health Perspect. 103, 268–271 7768228 10.1289/ehp.95103268 PMC1519064

[B40] Doval D. C. Pande S. B. Sharma J. B. Rao S. A. Prakash N. Vaid A. K. (2007). Report of a case of pericardial mesothelioma with liver metastases responding well to pemetrexed and platinum-based chemotherapy. J. Thorac. Oncol. 2, 780–781 10.1097/JTO.0b013e31811f3acd 17762349

[B41] Durant S. T. (2012). Telomerase-independent paths to immortality in predictable cancer subtypes. J. Cancer 2012, 3, 67–82. 10.7150/jca.3965 22315652 PMC3273709

[B42] Eilber F. C. Dry S. M. (2008). Diagnosis and management of synovial sarcoma. J. Surg. Oncol. 97, 314–320 10.1002/jso.20974 18286474

[B43] Erdogan E. Demirkazik F. B. Gulsun M. Ariyurek M. Emri S. Sak S. D. (2005). Incidental localized (solitary) mediastinal malignant mesothelioma. Br. J. Radiol. 78, 858–861 10.1259/bjr/19513813 16110113

[B44] Eren N. T. Akar A. R. (2002). Primary pericardial mesothelioma. Curr. Treat. Options Oncol. 3, 369–373 10.1007/s11864-002-0002-7 12194802

[B45] Finch A. Shaw P. Rosen B. Murphy J. Narod S. A. Colgan T. J. (2006). Clinical and pathologic findings of prophylactic salpingo-oophorectomies in 159 BRCA1 and BRCA2 carriers. Gynecol. Oncol. 100, 58–64 10.1016/j.ygyno.2005.06.065 16137750

[B46] Fox H. (1993). Primary neoplasia of the female peritoneum. Histopathol. 23, 103–110 10.1111/j.1365-2559.1993.tb00467.x 8406381

[B47] Fraire A. E. Cooper S. Greenberg S. D. Buffler P. Langston C. (1988). Mesothelioma of childhood. Cancer 62, 838–847 10.1002/1097-0142(19880815)62:4<838::AID-CNCR2820620433>3.0.CO;2-9 3293765

[B48] Frank T. S. (2001). Hereditary cancer syndromes. Arch. Pathol. Lab. Med. 125, 85–90 11151059 10.5858/2001-125-0085-HCS

[B49] Fujiwara H. Kamimori T. Morinaga K. Takeda Y. Kohyama N. Miki Y. (2005). An autopsy case of primary pericardial mesothelioma in arc cutter exposed to asbestos through talc pencils. Indust. Health 43, 346–350 10.2486/indhealth.43.346 15895852

[B50] Gaertner E. Zeren E. H. Fleming M. V. Colby T. V. Travis W. D. (1996). Biphasic synovial sarcoma arising in the pleural cavity. A clinicopathologic study of five cases. Am. J. Surg. Pathol. 20, 36–45 10.1097/00000478-199601000-00004 8540607

[B51] Galetta D. Pelosi G. Leo F. Solli P. Veronesi G. Borri A. (2007). Primary thoracic synovial sarcoma: factors affecting long-term survival. J. Thoracic Cardiovasc. Surg. 134, 808–809 10.1016/j.jtcvs.2007.05.036 17723844

[B52] Geisler J. P. Hatterman-Zogg M. A. Rathe J. A. Buller R. E. (2002). Frequency of BRCA1 dysfunction in ovarian cancer. J. Natl. Cancer. Inst. 94, 61–67 10.1093/jnci/94.1.61 11773283

[B53] Gibbs G. W. Berry G. (2008). Mesothelioma and asbestos. Regul. Toxicol. Pharmacol. 52, S223–S231 10.1016/j.yrtph.2007.10.003 18022298

[B54] Gilks C. B. Ionescu D. N. Kalloger S. E. Kobel M. Irving J. Clarke B. (2008). Tumor cell type can be reproducibly diagnosed and is of independent prognostic significance in patients with maximally debulked ovarian carcinoma. Human Pathol. 39, 1239–1251 10.1016/j.humpath.2008.01.003 18602670

[B55] Gladish G. W. Sabloff B. M. Munden R. F. Truong M. T. Erasmus J. J. Chasen M. H. (2002). Primary thoracic sarcomas. Radiographics 22, 621–637 10.1148/radiographics.22.3.g02ma17621 12006691

[B56] Gocha A. R. S. Harris J. Groden J. (2013). Alternative mechanisms of telomere lengthening: permissive mutations, DNA repair proteins, and tumorigenic progression. Mutat. Res. 743–744, 142–150 10.1016/j.mrfmmm.2012.11.006 23219603 PMC3619008

[B57] Gofman A. Issakov J. Kollender Y. Soyfer V. Dadia S. Jiveliouk I. (2007). Synovial sarcoma of the extremities and trunk: a long-lasting disease. Oncol. Rep. 18, 1577–1581 10.3892/or.18.6.1577 17982647

[B58] Goodman M. Teta M. J. Hessel P. A. Garabrant D. H. Craven V. A. Scrafford C. G. (2004). Mesothelioma and lung cancer among motor vehicle mechanics: a meta analysis. Ann. Occup. Hyg. 48, 309–326 10.1093/annhyg/meh022 15148053

[B59] Goyal R. K. (2005). Diseases of the esophagus, in Harrison's Principles of Internal Medicine, 16th Edn., eds Kasper D. L. (McGraw-Hill, New York), 1739–1762

[B60] Hankinson S. E. Danforth K. N. (2006). Ovarian Cancer, in Cancer Epidemiology and Prevention, 3rd Edn., eds Schottenfeld D. Fraumeni J. F. Jr (New York, NY: Oxford University Press). 1013–1026 10.1093/acprof:oso/9780195149616.003.0052

[B61] Harding A. H. Darnton A. J. (2010). Asbetsosis and mesothelioma among British asbestos workers (1971–2005). Am. J. Ind. Med. 53, 1070–1080 10.1002/ajim.20844 20957726

[B62] Hawley A. T. Pandolfi P. P. (2008). Etiology of cancer: cancer susceptibility syndromes, in Cancer, Principles and Practices of Oncology, 8th Edn., eds. DeVita V. T. D Lawrence T. S. S. A. Rosenberg (Philadelphia; Lippincott, Williams and Wilkins), 157–168

[B63] Heaphy C. M. de Wilde R. F. Jiao Y. Klein A. P. Edil B. H. Shi C. (2011). Altered telomeres in tumors with ATRX and DAXX mutations. Science 333:425 10.1126/science.1207313 21719641 PMC3174141

[B65] Hemminki K. Li X. (2003). Time trends and occupational risk factors for peritoneal mesothelioma in Sweden. J. Occup. Environ. Med. 45, 451–455 10.1097/01.jom.0000052960.59271.d4 12708149

[B64] Hernandez E. Bhagavan B. S. Parmley T. H. Rosenshein N. B. (1984). Interobserver variability in the interpretation of epithelial ovarian cancer. Gynecol. Oncol. 17, 117–123 10.1016/0090-8258(84)90065-9 6693048

[B66] Hillard A. K. Lovett J. K. McGavin C. R. (2003). The rise and fall in incidence of malignant mesothelioma from a British naval dockyards, 1979–1999. Occup. Med 53, 209–212 10.1093/occmed/kqg051 12724555

[B67] Hilton J. L. Geisler J. P. Rathe J. A. Hattermann-Zogg M. A. DeYoung B. Buller R. E. (2002). Inactivation of BRCA1 and BRCA2 in ovarian cancer. J. Natl. Cancer Inst. 94, 1396–1406 10.1093/jnci/94.18.1396 12237285

[B68] Hirano H. Maeda T. Tsuji M. Ito Y. Kizaki T. Yoshii Y. (2002). Malignant mesothelioma of the pericardium: case reports and immunohistochemical studies including Ki-67 expression. Pathol. Int. 52, 669–676 10.1046/j.1440-1827.2002.01404.x 12445141

[B69] Hodgson J. T. Darnton A. (2000). The quantitative risks of mesothelioma and lung cancer in relation to asbestos exposure. Ann. Occup. Hyg. 44, 565–601 10.1093/annhyg/44.8.565 11108782

[B172] Hodgson J. T. Darnton A. (2010). Mesothelioma from chrysotile. Occup. Environ. Med. 67, 432 10.1136/oem.2009.052860 19906656

[B70] Hodgson J. T. McElvenny D. M. Darnton A. J. Price M. J. Peto J. (2005). The expected burden of mesothelioma mortality in Great Britian from 2002 to 2050. Brit. J. Cancer 92, 587–593 10.1038/sj.bjc.6602307 15668716 PMC2362088

[B71] Hosono T. Hironaka M. Kobayashi A. Yamasawa H. Bando M. Ohno S. (2005). Primary pulmonary synovial sarcoma confirmed by molecular detection of SYT-SSX1 fusion gene transcripts: a case report and review of the literature. Jpn. J. Clin. Oncol. 35, 274–279 10.1093/jjco/hyi073 15879502

[B72] Hu J. Hwang S. S. Liesa M. Gan B. Sahin E. Jasekiloff M. (2012). Antitelomerase therapy provokes ALT and mitochondrial adaptive mechanisms in cancer. Cell 148, 651–663 10.1016/j.cell.2011.12.028 22341440 PMC3286017

[B73] Hubbard R. (1997). The aetiology of mesothelioma: are risk factors other than asbestos exposure important? Thorax 52, 496–497 10.1136/thx.52.6.496 9227712 PMC1758579

[B74] Ivanov S. V. Goparaju C. M. V. Lopez P. Zavadil J. Toren-Haritan G. Rosenwald S. (2010). Pro-tumorigenic effects of miR-31 loss in mesothelioma. J. Biol. Chem. 285, 22809–22817 10.1074/jbc.M110.100354 20463022 PMC2906272

[B75] Iwata T. Nishiyama N. Izumi N. Tsukioka T. Suehiro S. (2007). Metastatic monophasic synovial sarcoma of the pleura. Ann. Thoracic Cardiovasc. Surg. 13, 258–261 17717503

[B76] Jasani B. Gibbs A. (2012). Mesothelioma no associated with asbestos exposure. Arch. Pathol. Lab. Med. 136, 262–267 10.5858/arpa.2011-0039-RA 22372902

[B77] Jean D. Daubriac J. Le Pimpec-Barthes F. Galateau-Salle F. Jaurand M-C. (2012). Molecular changes in mesothelioma with an impact on prognosis and treatment. Arch. Pathol. Lab. Med. 136, 277–293 10.5858/arpa.2011-0215-RA 22372904

[B79] Kahn E. I. Rohl A. Barrett E. W. Suzuki Y. Suzuki Y. (1980). Primary pericardial mesothelioma following exposure to asbestos. Environ. Res. 23, 270–281 10.1016/0013-9351(80)90061-4 7472312

[B78] Kannerstein M. Churg J. McCaughey W. T. Hill D. P. (1977). Papillary tumors of the peritoneum in women: mesothelioma or papillary carcinoma. Am. J. Obstet. Gynecol. 127, 306–314 835626 10.1016/0002-9378(77)90475-6

[B80] Katabuchi H. Okamura H. (2003). Cell biology of human ovarian surface epithelial cells and ovarian carcinogenesis. Med. Electron Microsc. 36, 74–86 12886939 10.1007/s00795-002-0196-6

[B81] Kindelberger D. W. Lee Y. Miron A. Hirsch M. S. Feltmate C. Medeiros F. (2007). Intraepithelial carcinoma of the fimbria and pelvic serous carcinoma: evidence for a causal relationship. Am. J. Surg. Pathol. 31, 161–169 10.1097/01.pas.0000213335.40358.47 17255760

[B82] Kobel M. Kalloger S. E. Boyd N. McKinney S. Mehl E. Palmer C. (2008). Ovarian carcinoma subtypes are different diseases: implications for biomarker studies. PLoS Med. 5:e232 10.1371/journal.pmed.0050232 19053170 PMC2592352

[B83] Kolschmann S. Ballin A. Gillissen A. (2005). Thoracoscopic talc pleurodesis in malignant pleural effusions. Chest 128, 1431–1437 10.1378/chest.128.3.1431 16162739

[B84] Kumar V. Abbas A. K. Fausto N. (2005a). Germ cell tumors, in Robbins and Cotran Pathologic Basis of Disease, 7th Edn, eds Kumar V. Abbas A. K. Fausto N. (Philadelphia: Elsevier Saunders), 1040–1048

[B185] Kumar V. Abbas A. K. Fausto N. (2005b). Congenital heart defects, in Robbins and Cotran Pathologic Basis of Disease, 7th Edn, eds Kumar V. Abbas A. K. Fausto N. (Philadelphia: Elsevier Saunders), 564–571

[B186] Kumar V. Abbas A. K. Fausto N. (2005c). Synovial sarcoma, in Robbins and Cotran Pathologic Basis of Disease, 7th Edn, eds Kumar V. Abbas A. K. Fausto N. (Philadelphia: Elsevier Saunders), 1323–1324

[B85] Laden F. Stampfer M. J. Walker A. M. (2004). Lung cancer and mesothelioma among male automobile mechanics: a review. Rev. Environ. Health 19, 39–61 10.1515/REVEH.2004.19.1.39 15186039

[B86] Lagrotteria D. D. Tsang B. Elavathil L. J. Tomlinson C. W. (2005). A case of primary malignant pericardial mesothelioma. Can. J. Cardiol. 21, 185–187 15729420

[B87] Larson T. Melinkova N. Davis S. I. Jamison P. (2007). Incidence and descriptive epidemiology of mesothelioma in the United states, 1999–2002. Int. J. Occup. Environ. Health 13, 398–403 10.1179/oeh.2007.13.4.398 18085053

[B88] Lauchlan S. C. (1972). The secondary mullerian system. Obstet. Gynecol. Surv. 27, 133–146 10.1097/00006254-197203000-00001 4614139

[B89] Lee H. K. Kwon H. J. Lee H. B. Jin G. Y. Chung M. J. Lee Y. C. (2006). Radiofrequency thermal ablation of primary pleural synovial sarcoma. Respiration 73, 250–252 10.1159/000087153 16043956

[B90] Lee Y. Miron A. Drapkin R. Nucci M. R. Medeiros F. Saleemuddin A. (2007). A candidate precursor to serous carcinoma that originates in the distal fallopian tube. J. Pathol. 211, 26–35 10.1002/path.2091 17117391

[B91] Leigh J. (2003). Letter to USEPA from J. Leigh with Subject: 1986 EPA “Gold Book” Guidance for Preventing Asbestos-Related Disease Among Auto Mechanics. Request for correction, available at #12, University of Sydney

[B92] Lemmon M. A. Schlessinger J. (2010). Cell signaling by receptor tyrosine kinases. Cell 14, 1117–1134 10.1016/j.cell.2010.06.011 20602996 PMC2914105

[B93] Levine D. A. Argenta P. A. Yee C. J. Marshall D. S. Olvera N. Bogomolniy F. (2003). Fallopian tube and primary peritoneal carcinomas associated with BRCA mutations. J. Clin. Oncol. 21, 4222–4227 10.1200/JCO.2003.04.131 14615451

[B94] Luk A. Ahn E. Vaideeswar P. Butany J. W. (2008). Pericardial tumors. Semin. Diagnos. Pathol. 25, 47–53 10.1053/j.semdp.2007.12.001 18350922

[B95] Lund B. Thomsen H. K. Olsen J. (1991). Reproducibility of histopathological evaluation in epithelial ovarian carcinoma. Clinical implications. APMIS 99, 353–358 10.1111/j.1699-0463.1991.tb05161.x 2036219

[B96] Magnani C. Ferrante D. Barone-Adesi F. Bertolotti M. Mirabelli D. Terracini B. (2007). Cancer risk after cessation of asbestos exposure, a cohort study of Italian asbestos cement workers. Occup. Environ. Med. 31, 16–22 10.1136/oem.2007.032847 17704197

[B97] Makhlouf H. R. Ahrens W. Agarwal B. Dow N. Marshalleck J. J. Lee E. L. (2008). Synovial sarcoma of the stomach: a clinicopathologic, immunohistochemical, and molecular genetic study of 10 cases. Am. J. Surg. Pathol. 37, 275–281 10.1097/PAS.0b013e31812e6a58 18223331

[B98] Martini V. Michelazzi L. Cioe A. Fucile C. Spigno F. Robbiano L. (2011). Exposure to asbestos: correlation between blood levels of mesothelin and frequency of micronuclei in peripheral blood lymphocytes. Mutat. Res. 721, 114–117 10.1016/j.mrgentox.2010.12.014 21238604

[B99] Maruyama R. Sakai M. Nakamura T. Suemitsu R. Okamoto T. Wataya H. (2006). Triplet chemotherapy for malignant pericardial mesothelioma: a case report. Jpn. J. Clin. Oncol. 36, 245–248 10.1093/jjco/hyl003 16533802

[B100] Mayer R. L. (2005). Gastrointestinal tract cancer, in Harrison's Principles of Internal Medicine, 16th Edn., eds Kasper D. L. (New York, NY: McGraw-Hill), 523–533

[B101] McCoubrey A. Houghton O. McCallion K. McCluggage W. G. (2005). Serous adenocarcinoma of the sigmoid mesentery arising in cystic endosalpingiosis. J. Clin. Pathol. 58, 1221–1223 10.1136/jcp.2005.027946 16254118 PMC1770769

[B102] Medeiros F. Muto M. G. Lee Y. Elvin J. A. Callahan M. J. Feltmate C. (2006). The tubal fimbria is a preferred site for early adenocarcinoma in women with familial ovarian cancer syndrome. Am. J. Surg. Pathol. 30, 230–236 10.1097/01.pas.0000180854.28831.77 16434898

[B103] Michal M. Fanburg-Smith J. C. Lasota J. Fetsch J. F. Lichy J. Mettinen M. (2006). Minute synovial sarcomas of the hands and feet: a clinicopathologic study of 21 tumors less than 1 cm. Am. J. Surg. Pathol. 30, 721–726 10.1097/00000478-200606000-00007 16723849

[B104] Miettinen M. Limon J. Niezabitowski A. Lasota J. (2001). Calretinin and other mesothelioma markers in synovial sarcoma: analysis of antigentic similarities and differences with malignant mesothelioma. Am. J. Surg. Pathol. 25, 610–617 10.1097/00000478-200105000-00007 11342772

[B105] Mizell K. N. Morris C. G. Carter J. E. (2009). Antemortem diagnosis of asbestosis by screening chest radiograph correlated with postmortem histologic features of asbestosis: a study of 273 cases. J. Occup. Med. Toxicol. 4:14 10.1186/1745-6673-4-14 19523203 PMC2704219

[B106] Molina Garrido M. J. Rufete A. M. Rodriguez-Lescure A. Cascón Pérez J. D. Guillén Ponce C. Carrato Mena A. (2006). Recurrent pericardial effusion as initial manifestation of primary diffuse pericardial malignant mesothelioma. Clin. Transl. Oncol. 8, 694–696 10.1007/s12094-006-0042-8 17005474

[B174] Moolgavkar S. H. Mesa R. Turim J. (2009). Pleural and peritoneal mesotheliomas in SEER: age effects and temporal trends, 1973–2005. Cancer Causes Control 20, 935–944 10.1007/s10552-009-9328-9 19294523

[B107] Moran C. A. Suster S. Koss M. N. (1992). The spectrum of histologic growth patterns in benign and malignant fibrous tumors of the pleura. Semin. Diagnost. Pathol. 9, 169–180 1609159

[B108] Morin P. J. Trent J. M. Collins F. S. Vogelstein B. (2005). Cancer genetics, in Harrison's Principles of Internal Medicine, 16th Edn., eds Kasper et al. D. L. (New York, NY: McGraw-Hill), 447–453

[B109] Motzer R. J. Bosl G. J. (2005). Testicular Cancer, in Harrison's Principles of Internal Medicine, 16th Edn., eds Kasper D. L. Braunwald E. Hauser S. Longo D. Jameson J. L. Fauci A. S. (New York, NY: McGraw-Hill), 550–553

[B110] Mukhopadhyay S. Aubry M. C. (2007). Recurrent primary synovial sarcoma of the chest wall. J. Thorac. Oncol. 2, 660–661 10.1097/JTO.0b013e31807a2f99 17607124

[B111] Muto M. G. (2005). The patient at risk of ovarian cancer, in Diagnostic gynecologic and Obstetric Pathology, eds Crum C. P. Lee K. R. (New York, NY: Elsevier Saunders), 811–819

[B112] Neri M. Filiberti R. Taioli E. Garte S. Paracchini V. Bolognesi C. (2005). Pleural malignant mesothelioma, genetic susceptibility and asbestos exposure. Mutat. Res. 592, 36–44 10.1016/j.mrfmmm.2005.06.003 15993904

[B113] Neumann V. Gunther S. Muller K.-M. Fischer M. (2001). Malignant mesothelioma—German mesothelioma register 1987–1999. Int. Arch. Occup. Environ. Health 74, 383–395 10.1007/s004200100240 11563601

[B114] Nicholson A. G. Goldstraw P. Fisher C. (1998). Synovial sarcoma of the pleura and its differentiation from other primary pleural tumours: a clinicopathological and imuunohistochemical review of three cases. Histopathology 33, 508–513 10.1046/j.1365-2559.1998.00565.x 9870144

[B115] Niggli F. K. Gray T. J. Raafat F. Stevens M. C. (1994). Spectrum of peritoneal mesothelioma in childhood: clinical and histopathologic features, including DNA cytometery. Pediatr. Hematol. Oncol. 11, 399–408 10.3109/08880019409140539 7947012

[B116] Ordonez N. G. (1998). In search of a positive immunohistochemical marker for mesothelioma: an update. Adv. Anat. Pathol. 5, 53–60 10.1097/00125480-199801000-00051 9868512

[B117] Paley P. J. Swisher E. M. Garcia R. L. Agoff S. N. Greer B. E. (2001). Occult cancer of the fallopian tube in BRCA-1 germline mutation carriers at prophylactic oophorectomy: a case for recommending hysterectomy at surgical prophylaxis. Gynecol. Oncol. 80, 176–180 10.1006/gyno.2000.6071 11161856

[B118] Papi M. Genestreti G. Tassinari D. Lorenzini P. Serra S. Ricci M. (2005). Malignant pericardial mesothelioma. Report of two cases, review of the literature and differential diagnosis. Tumori 91, 276–279 16206657 10.1177/030089160509100315

[B119] Parmley T. H. Woodruff J. D. (1974). The ovarian mesothelioma. Am. J. Obstet. Gynecol. 120, 234–241 4416395 10.1016/0002-9378(74)90370-6

[B120] Pass H. I. Hahn S. M. Vogelzang N. J. Carbone M. (2005). Benign and malignant mesothelioma, in Cancer, Principles and Practice of Oncology, 8th Edn., eds DeVita V. T. Lawrence T. S. Rosenberg S. A. (Philadelphia: Lippincottt, Williams and Wilkins), 1687–1750 10.1007/0-387-28274-2

[B121] Pass H. I. Vogelzang N. T. Hahn S. M. Carbone M. (2008). Benign and malignant mesothelioma, in Cancer, Principles And Practice of Oncology, 8th Edn., eds. DeVita V. T. Lawrence T. S.L Rosenberg S. A. R (Philadelphia: Lippincottt, Williams and Wilkins), 1835–1862

[B122] Piek J. M. Kenemans P. Verheijen R. H. M. (2004). Intraperitoneal serous adenocarcinoma: a critical appraisal of three hypotheses on its cause. Am. J. Obstet. Gynecol. 191, 718–732 10.1016/j.ajog.2004.02.067 15467531

[B123] Piek J. M. Torrenga B. Hermsen B. Verheijen R. H. Zweemer R. P. Gille J. J. (2003). Histopathological characteristics of BRCA1- and BRCA2—associated intraperitoneal cancer: a clinic—based study. Fam. Cancer 2, 73–78 10.1023/A:1025700807451 14574155

[B124] Pisick E. Salgia R. (2005). Molecular biology of malignant mesothelioma: a review. Hematol. Oncol. Clin. North Am. 19, 997–1023 10.1016/j.hoc.2005.09.012 16325120

[B125] Piura B. Rabinovich A. Yanai-Inbar I. (2001). Three primary malignancies related to BRCA mutation successively occurring in a BRCA1 185delAG mutation carrier. Eur. J. Obstet. Oncol. Gynecol. Reprd. Biol. 97, 241–244 10.1016/S0301-2115(00)00521-2 11451557

[B126] Powell C. B. Kenley E. Chen L. M. Crawford B. McLennan J. Zaloudek C. (2005). Risk—reducing Salpingo—Oophorectomy in BRCA mutation carriers: role of serial sectioning in the detection of occult malignancy. J. Clin. Oncol. 23, 127–132 10.1200/JCO.2005.04.109 15625367

[B127] Price B. Ware A. (2004). Mesothelioma trends in the United States: an update based on surveillance, epidemiology, and end results program data for 1973 through 2003. Am. J. Epidemiol. 159, 107–112 10.1093/aje/kwh025 14718210

[B128] Quinn D. W. Qureshi F. Mitchell I. M. (2000). Pericardial mesothelioma: the diagnostic dilemma of misleading images. Ann. Thoracic Surg. 69, 1926–1927 10.1016/S0003-4975(00)01204-2 10892948

[B129] Raju U. Fine G. Greenawald K. A. Ohorodnik J. M. (1989). Primary papillary serous neoplasia of the peritoneum: a clinicopathologic and ultrastructural study of eight cases. Human Pathol. 20, 426–436 10.1016/0046-8177(89)90006-3 2707793

[B130] Rdzanek M. Fresco R. Pass H. I. Carbone M. (2006). Spindle cell tumors of the pleura: differential diagnosis. Semin. Diagnost. Pathol. 23, 44–55 10.1053/j.semdp.2006.06.002 17044195

[B131] Reid A. de Klerk N. Ambrosini G. Olsen N. Pang S. C. Musk A. W. (2005). The additional risk of malignant mesothelioma in former workers and residents of Witenoom with benign pleural disease or asbestosis. Occup. Environ. Med 62, 665–669 10.1136/oem.2004.018531 16169910 PMC1740875

[B132] Reis-Filho J. S. Pope L. Z. B. Milanezi F. Balderrama C. M. Serapião M. J. Schmitt F. C. (2002). Primary epithelial malignant mesothelioma of the pericardium with deciduoid features: cytohistologic and immunohistochemical. Diagnost. Cytopathol. 26, 117–122 10.1002/dc.10068 11813331

[B133] Risch H. A. McLaughlin J. R. Cole D. E. Rosen B. Bradley L. Kwan E. (2001). Prevalence and penetrance of germline BRCA1 and BRCA2 mutations in a population series of 649 women with ovarian cancer. Am. J. Hum. Genet. 68, 700–710 10.1086/318787 11179017 PMC1274482

[B134] Robinson B. W. S. Creaney J. Lake R. Nowak A. Musk A. W. de Klerk N. (2003). Mesothelin-family proteins and diagnosis of mesothelioma. Lancet 362, 1612–1616 10.1016/S0140-6736(03)14794-0 14630441

[B135] Sakamoto A. Sasaki H. Furusato M. Suzuki M. Hirai Y. Tsugane S. (1994). Observer disagreement in histological classification of ovarian tumors in Japan. Gynecol. Oncol. 54, 54-58 10.1006/gyno.1994.1165 8020839

[B136] Sandberg A. A. (2002). Cytogenetics and molecular genetics of bone and soft tissue tumors. Am. J. Med. Genet. 115, 189–193 10.1002/ajmg.10691 12407700

[B137] Santos C. Montesinos J. Castaner E. Sole J. M. Baga R. (2008). Primary pericardial mesothelioma. Lung Cancer 60, 291–293 10.1016/j.lungcan.2007.08.029 17936406

[B138] Sarma A. V. McLaughlin J. C. Schottenfeld D. (2006). Testicular cancer, in Cancer Epidemiology and Prevention, 3rd Edn, eds Schottenfeld D. Fraumeni J. F. (New York, NY: Oxford University Press), 1151–1165 10.1093/acprof:oso/9780195149616.003.0060

[B139] Satoh H. Ohara G. Hizawa N. (2007). Primary synovial sarcoma of the chest wall. J. Thorac. Oncol. 2:1060 10.1097/JTO.0b013e318158ef37 17975502

[B140] Scherpereel A. Grigoriu B. Conti M. Gey T. Gregoire M. Copin M.-C (2006). Soluble mesothelin-related peptides in the diagnosis of malignant pleural mesothelioma. Am. J. Respir. Crit. Care Med. 173, 1155–1160 10.1164/rccm.200511-1789OC 16456138

[B141] Schorge J. O. Miller Y. B. Qi L. J. Muto M. G. Welch W. R. Berkowitz R. S. (2000). Genetic alterations of the WT1 gene in papillary serous carcinoma of the peritoneum. Gynecol. Oncol. 76, 369–372 10.1006/gyno.1999.5711 10684712

[B142] Shimizu M. Toki T. Takagi Y. Konishi I. Fuji S. (2000). Immunohistochemical detection of the Wilms' tumor gene (WT1) in epithelial ovarian tumors. Int. J. Gynecol. Pathol. 19, 158–163 10.1097/00004347-200004000-00010 10782413

[B143] Shiraki M. Enterline H. T. Brooks J. J. Cooper N. S. Hirschi S. Roth J. A. (1989). Pathologic analysis of advanced adult soft tissue sarcomas, bone sarcomas, and mesotheliomas. The Eastern Cooperative Oncology Group (ECOG) experience. Cancer 64, 484–490 10.1002/1097-0142(19890715)64:2<484::AID-CNCR2820640223>3.0.CO;2-T 2736494

[B144] Sichletidis L. Chloros D. Spyratos D. Haidich A. B. Fourkiotou I. Kakoura M. (2008). Mortality from occupational exposure to relatively pure chrysotile: a 39-year study. Respiration 78, 63–68 10.1159/000163443 18843176

[B145] Siegel H. J. Sessions W. Casillas M. A. Said-Al-Naief N. Lander P. H. Lopez-Ben R. (2007). Synovial sarcoma: clinicopathologic features, treatment, and prognosis. Orthopedics 30, 1020–1025 Available online at: http://www.healio.com/orthopedics/oncology/journals/ortho/%7Bcfaffd56-2438-49b0-918e-551612fc369a%7D/synovial-sarcoma-clinicopathologic-features-treatment-and-prognosis 18198773 10.3928/01477447-20071201-15

[B146] Sobol H. Jacquemier J. Bonaiti C. Dauplat J. Birnbaum D. Eisinger F. (2000). Fallopian tube cancer as a feature of BRCA1-associated syndromes. Gynecol. Oncol. 78, 263–266 10.1006/gyno.2000.5897 10926816

[B147] Sporn T. A. Roggli V. L. (2004). Mesothelioma, in Pathology of Asbestos-Associated Diseases, 2nd Edn., eds. Roggli V. L. Oury T. D. Sporn T. A. S (New York, NY: Springer), 104–168 10.1007/0-387-21819-X_5

[B148] Struewing J. P. Harge P. Wacholder S. Baker S. M. Berlin M. McAdams M. (1997). The risk of cancer associated with specific mutations of BRCA1 and BRCA2 among Ashkenazi Jews. New Eng. J. Med. 336, 1401–1408 10.1056/NEJM199705153362001 9145676

[B149] Susman S. Schofield P. Large S. (2004). Primary pericardial mesothelioma presenting as pericardial constriction: a case report. Heart 90:e4 10.1136/heart.90.1.e4 14676267 PMC1767997

[B150] Tallet A. Nault J-C. Renier A. Hysi I. Galateau-Salle F. Cazes A. (2013). Overexpression and promoter mutation of the TERT gene in malignant pleural mesothelioma. Oncogene. . [Epub ahead of print]. 10.1038/onc.2013.351 23975423

[B151] Taylor C. A. Barnhart A. Pettenati M. J. Geisinger K. R. (2005). Primary pleuropulmonary synovial sarcoma diagnosed by fine needle aspiration with cytogenetic confirmation: a case report. Acta Cytol. 49, 673–676 10.1159/000326260 16450912

[B152] Testa J. R. Cheung M. Pei J. Below J. E. Tan Y. Sementino E. (2011). Germline BAP1 mutations predispose to malignant mesothelioma. Nat. Genetics 43, 1022–1026 10.1038/ng.912 21874000 PMC3184199

[B153] Teta M. J. Mink P. J. Lau E. Sceurman B. K. Foster E. D. (2008). U.S. mesothelioma patterns 1973–2002: indicators of change and insights into background rates. Eur. J. Cancer Prev. 17, 525–534 10.1097/CEJ.0b013e3282f0c0a2 18941374

[B154] Tewari S. C. Kurian G. Jayaswal R. Chakravorty S. Chadha S. K. Chauhan M. S. (1989). Malignant mesothelioma in the young (with prosthetic aortic valve, an unusual association). J. Assoc. Phys. India 37, 187–189 2808292

[B155] Thomason R. Schlegel W. Lucca M. Cummings S. Lee S. (1994). Primary malignant mesothelioma of the pericardium. Case report and literature review. Tex. Heart Inst. J. 21, 170–174 8061543 PMC325154

[B156] Tsukioka T. Inoue K. Iwata T. Mizuguchi S. Morita R. Suehiro S. (2006). Resected case of synovial sarcoma in the pleural cavity. Jpn. J. Thorac. Cardiovasc. Surg. 54, 263–266 10.1007/PL00022249 16813111

[B157] Val-Bernal J. F. Figols J. Gomez-Roman J. J. (2002). Incidental localized (solitary) epithelial mesothelioma of the pericardium case report and literature review. Cardiovasc. Pathol. 11, 181–185 10.1016/S1054-8807(02)00097-2 12031772

[B158] Vera J. Garcia M. D. Marigil M. Abascal M. Lopez J. I. Ligorred L. (2006). Biphasic synovial sarcoma of the abdominal wall. Virchows Arch. 449, 367–372 10.1007/s00428-005-0076-2 16855839

[B159] Vohra H. A. Davies S. Vohra H. Rosin M. D. Snead D. R. (2004). Primary synovial sarcoma of the pleura: beware of misdiagnosis. Eur. J. Intern. Med. 15, 465–466 10.1016/j.ejim.2004.08.004 15581753

[B160] Vornicu M. Arora S. Achilleos A. (2007). Primary pericardial mesothelioma: a rare cardiac malignancy. Intern. Med. J. 37, 576–577 10.1111/j.1445-5994.2007.01409.x 17640193

[B161] Weill H. Hughes J. M. Churg A. M. (2004). Changing trends in US mesothelioma incidence. Occup. Environ. Med. 61, 438–441 10.1136/oem.2003.010165 15090665 PMC1740785

[B162] Weinbreck N. Vignaud J. M. Begueret H. Burke L. Benhattar J. Guillou L. (2007). SYT-SSX fusion is absent in sarcomatoid mesothelioma allowing its distinction from synovial sarcoma of the pleura. Modern Pathol. 20, 617–621 10.1038/modpathol.3800775 17507990

[B163] Werness B. A. Ramus S. J. Whittemore A. S. Garlinghouse-Jones K. Oakley-Girvan I. Dicioccio R. A. (2000). Histopathology of familial ovarian tumors in women from families with and without germline BRCA1 mutations. Hum. Pathol. 31, 1420–1424 10.1016/S0046-8177(00)80014-3 11112219

[B164] Winston C. B. Hadar O. Teitcher J. B. Caravelli J. F. Sklarin N. T. Panicek D. M. (2000). Metastatic lobular carcinoma of the breast: patterns of spread in the chest, abdomen, and pelvis on CT. Am. J. Roentgenol. 175, 795–800 10.2214/ajr.175.3.1750795 10954469

[B165] Yakirevich E. Sova Y. Drumea K. Bergman I. Quitt M. Resnick M. B. (2004). Peripheral lymphadenopathy as the initial manifestation of pericardial mesothelioma: a case report. Int. J. Surg. Pathol. 12, 403–405 10.1177/106689690401200415 15494868

[B166] Yarborough C. M. (2006). Chrysotile as a cause of mesothelioma: an assessment based on epidemiology. Off. Rev. Toxicol. 36, 165–187 10.1080/10408440500534248 16736942

[B177] Yarborough C. M. (2007). The risk of mesothelioma from exposure to chrysotile asbestos. Curr. Opin. Pulm. Med. 13, 334–338 10.1097/MCP.0b013e328121446c 17534182

[B167] Zweemer R. P. van Diest P. J. Verheijen R. H. Ryan A. Gille J. J. Sijmons R. H. (2000). Molecular evidence linking primary cancer of the fallopian tube to BRCA1 germline mutations. Gynecol. Oncol. 76, 45–50 10.1006/gyno.1999.5623 10620440

